# Sulfur compounds block MCP-1 production by *Mycoplasma fermentans*-infected macrophages through NF-κB inhibition

**DOI:** 10.1186/1479-5876-12-145

**Published:** 2014-05-24

**Authors:** Francesca Benedetti, Sergio Davinelli, Selvi Krishnan, Robert C Gallo, Giovanni Scapagnini, Davide Zella, Sabrina Curreli

**Affiliations:** 1 Institute of Human Virology, University of Maryland School of Medicine, Baltimore, MD 21201, USA; 2 Department of Biomedical, Biotechnological and Translational Sciences (S.Bi.Bi.T.), Anatomy and Histology Unit, University of Parma, Ospedale Maggiore, 43100 Parma, Italy; 3 Department of Medicine and Health Sciences, University of Molise, Campobasso 86100, Italy

**Keywords:** Hydrogen sulfide (H_2_S), NaHS, GYY4137, MCP-1, Mycoplasma fermentans, Monocytes/macrophages, NF-κB

## Abstract

**Background and aims:**

Hydrogen sulfide (H_2_S), together with nitric oxide (NO) and carbon monoxide (CO), belongs to a family of endogenous signaling mediators termed “gasotransmitters”. Recent studies suggest that H_2_S modulates many cellular processes and it has been recognized to play a central role in inflammation, in the cardiovascular and nervous systems. By infecting monocytes/macrophages with *Mycoplasma fermentans* (M.F.), a well-known pro-inflammatory agent, we evaluated the effects of H_2_S.

**Methods:**

M.F.-infected cells were analyzed by ELISA and real time RT-PCR to detect the M.F. effects on MCP-1 and on MMP-12 expression. The role of two different H_2_S donors (NaHS and GYY4137) on MF-infected cells was determined by treating infected cells with H_2_S and then testing the culture supernatants for MCP-1 and on MMP-12 production by ELISA assay. In order to identify the pathway/s mediating H_2_S- anti-inflammatory activity, cells were also treated with specific pharmaceutical inhibitors. Cytoplasmic and nuclear accumulation of NF-κB heterodimers was analyzed.

**Results:**

We show that H_2_S was able to reduce the production of pro-inflammatory cytokine MCP-1, that was induced in monocytes/macrophages during M.F. infection. Moreover, MCP-1 was induced by M.F. through Toll-like receptor (TLR)-mediated nuclear factor-κB (NF-κB) activation, as demonstrated by the fact that TLR inhibitors TIRAP and MyD88 and NF-κB inhibitor IKK were able to block the cytokine production. In contrast H_2_S treatment of M.F. infected macrophages reduced nuclear accumulation of NF-κB heterodimer p65/p52.

**Conclusions:**

Our data demonstrate that under the present conditions H_2_S is effective in reducing Mycoplasma-induced inflammation by targeting the NF-κB pathway. This supports further studies for possible clinical applications.

## Background

Hydrogen sulfide (H_2_S), regarded for decades as a toxic gas, is a ubiquitous gas produced endogenously in the human body and is included in the family of gasotransmitters, together with nitric oxide (NO) and carbon monoxide (CO) [1]. H_2_S has been implicated in several physiological and pathological contexts including oxidative stress regulation via scavenging reactive oxygen species (ROS), inflammation, vasodilation and neuronal survival [2]. As a gaseous signaling molecule, H_2_S diffuse freely across cell membranes in a receptor-independent manner and activate various cellular targets. Its effects on the cell viability, proliferation, activation, cytokines secretion and cell adhesion have been investigated in many different cell types [3]. H_2_S has been widely demonstrated to have cardio-protective effects [4], pro-angiogenic [5] and vasorelaxing effects [6] both in *in vivo* and *in vitro* experiments. Also H_2_S mediates KATP channel opening [7], it has inhibitory effect on platelet aggregation [8] and anti-apoptotic [9] and cytoprotective effects [10].

The precise role of H_2_S in inflammation is still far from clear: in fact it may have pro- or anti- inflammatory effects under different conditions [11]. These discrepancies may reflect the varying effects of a dose–response relationship. Several studies have demonstrated that H_2_S donors, in addition to suppressing leukocyte adherence to the vascular endothelium and infiltration to the sites of inflammation [12], can reduce the expression of several pro-inflammatory cytokines. Indeed, H_2_S inhibits the activation of the transcription factor nuclear factor-κB (NF-κB), essential for the activation of most pro-inflammatory genes, in murine macrophages RAW264.7 cell line following exposure to bacterial endotoxin and blocks the increase of inducible nitric oxide synthase (iNOS) expression and NO production [13]. Moreover H_2_S inhibits IkB-α degradation and thereby NF-κB translocation to the nucleus in HUVEC cells stimulated with tumor necrosis factor-α (TNF-α) [14] and in astrocytes stimulated with LPS [15]. Similarly, H_2_S inhibits endotoxin-induced upregulation of iNOS expression, NO production and TNF-α expression in cultured microglia. These effects were attributed at least in part to the suppression by H_2_S of endotoxin-induced p38 mitogen-activated protein (MAP) kinase phosphorylation [16]. Administration of H_2_S to LPS-injected rats resulted in the activation of STAT3, which is known to regulate the expression of many genes that mediate cell survival, proliferation and angiogenesis [17]. Furthermore H_2_S administration induces the activation and the nuclear localization of the transcription factor NF-E2-related factor-2 (Nrf-2) in ischemic rat hearts [18]. Nrf-2 is a master regulator of antioxidant transcriptional responses with a protective role in the lungs, mediated through the activation of antioxidant and cytoprotective genes [19]. Moreover H_2_S increases NO production with consequential down-regulation of the pro-angiogenic cytokine VEGF (vascular endothelial growth factor) in human keratinocytes [20].

*Mycoplasma fermentans* (M.F.), which belongs to the Mollicutes class, is a self-replicating wall-less prokaryote, surrounded only by a plasma membrane and with limited metabolic capabilities [21,22]. M.F. has been associated with the onset and progression of several human pathologies [23], including chronic inflammatory diseases such as rheumatoid arthritis [24,25], respiratory and genitourinary tract infections [26]. M.F. pathogenesis is through sophisticated mechanisms for evasion of immune surveillance (molecular mimicry and a unique type of antigenic variation), up-regulating or down-regulating cytokines secretion, adhesion molecules and transcription factors expression, and MAP kinases activity [22,27]. M.F. induces the production of cytokines such as IL-1, IL-2, IL-4, IL-6, interferons, TNF-α and GM-CSF [28]. Although the immunomodulatory role of M.F. is well established, its pathogenic mechanisms remain mostly unknown.

Monocyte Chemoattractant Protein-1 (MCP-1), also known as CCL2, is a member of the C-C chemokine family and a potent chemotactic factor for monocytes. MCP-1 is produced by a variety of cell types and monocyte/macrophages are the major source of this chemokine [29]. MCP-1 mediates its effects through its receptors CCR2 and CCR4 and regulates the migration and infiltration of monocytes, memory T lymphocytes and natural killer cells [30]. In addition to its chemotactic activity for leukocytes, several line of evidence indicate that MCP-1 plays a role in tumor metastasis and angiogenesis, as well as in the modulation of cell proliferation, apoptosis and protein synthesis [31]. Of note, MCP-1 is a potential intervention point for the treatment of various diseases, including multiple sclerosis [32], rheumatoid arthritis [33], atherosclerosis [34] and insulin-resistant diabetes [35].

Monocytes/macrophages play a central role in the initiation and resolution of inflammation: they act principally through phagocytosis, release of pro-inflammatory cytokines and reactive oxygen species (ROS) and the activation of the acquired immune system. M.F. triggers rapid recruitment of a large number of macrophages especially into the lungs and airways, thus monocytes/macrophages play critical role in M.F. clearance [36].

There is a growing interest in “medical gasses” for their antibacterial and anti-inflammatory properties. In this study we investigated the effects of H_2_S in M.F. infection. Due to the relevance of monocytes/macrophages in inflammation and against M.F. infection [23,37], we used primary macrophages cultures and human monocytic cell line U937 as an *in vitro* model to investigate the effects of H_2_S on the expression of MCP-1 pro-inflammatory chemokine following M.F. infection.

## Methods

### Bacterial strains and culture conditions

*Mycoplasma fermentans* (M.F.) PG18 *(*American Type Culture Collection) was grown in 243 media: heart infusion broth (BD) media supplemented with 20% heat inactivated horse serum and 10% yeast extract solution (Invitrogen, Grand Island, NY, USA), at 37°C in aerobic conditions. Mycoplasma cultures were harvested in late log phase and collected by centrifugation (10 min at 10,000 × g at 4°C), and washed three times with PBS before using. Cells were infected with a concentration of 2 CFU/cell.

U937 cells were grown in RPMI 1640 Medium (Invitrogen, Grand Island, NY, USA) supplemented with 10% fetal bovine serum (FBS).

Peripheral blood mononuclear cells (PBMC) were isolated by Ficoll-Hystopaque density-gradient centrifugation (Sigma-Aldrich, St. Louis, MO, USA) of heparinized leukocyte units obtained from healthy donors purchased from the New York Blood Center. To obtain primary macrophages cultures, PBMC were seeded into 24-well plates at 3x10^6^ per well or 12-well plates at 5.7x10^6^ per well in RPMI 1640 medium (Invitrogen, Grand Island, NY, USA) supplemented with 20% FBS (Gemini Bio-Products, Burlington, Ontario, Canada) and 10% human serum (Gemini Bio-Products, Burlington, Ontario, Canada). Seven days later, when the macrophages monolayers were well established, the cultures were washed several times to remove non adherent cells, and then cultured in the medium described above but lacking human serum. Cultures were fed at 3 days intervals with a complete medium exchange.

### Cells treatments

Cells were treated with NaHS at 1 mM (Sigma-Aldrich, St. Louis, MO, USA) and GYY4137 at 100 μM (Santa Cruz Biotechnology, Dallas, TX, USA) final concentration, and the related controls: NaHCO_3_ (Sigma-Aldrich, St. Louis, MO, USA) for NaHS and PBS (Invitrogen, Grand Island, NY, USA) for GYY4137. Reagents were added directly into the culture medium. Optimal and non-toxic NaHS and GYY4137 concentration were determined by treating U937 with 0.02, 0.1, 0.5, 1 or 2 mM NaHS and GYY4137 for 24 hours. NaHS and GYY4137 range of concentrations was chosen in reference to the physiological level of H_2_S in the body. The cells viability was determined with propidium iodide (PI) (2.5 μg/ml) staining and analyzed by flow cytometry.

Cells were also treated with IKK Inhibitor VII (200 nM) and TIRAP Inhibitor peptide (10 μM) to induce NF-κB inhibition, MyD88 Inhibitory Peptide (75 μM) to inhibit the homodimerization of MyD88, the major adaptor molecule for all TLRs, and SB203580 (400 μM), an inhibitor of p38-MAP kinase. The correct concentrations were obtained by MTS assay (CellTiter 96 Aqueous One Solution Reagent – Promega). All inhibitors were from Calbiochem (EMD Millipore, Billerica, MA, USA) with the exception of MyD88 Inhibitory Peptide that was from Novus Biologicals (IMGENEX, San Diego, CA, USA). Equal volumes of DMSO or MyD88 control peptide were added to control samples. All inhibitors were added to the culture medium every 24 h at pre-determined optimal and non-toxic concentrations (data not shown).

### Cytokines assay

Bio-Plex Pro™ human cytokine 27-plex immunoassay (BioRad, Hercules, CA, USA) was used to detect the following cytokines and chemokines: IL-1β, IL-1α, IL-2, IL-4, IL-5, IL-6, IL-7, IL-8, IL-9, IL-10, IL-12, IL-13, IL-15, IL-17A, IL-18, Eotaxin, basic FGF, G-CSF, GM-CSF, IFN-γ, IP-10, MCP-1, MIP-1α, MIP-1β, RANTES, TNF-α, PDGF, VEGF, CTACK, GROα, HGF, IFNα2, MCP-3, MIF, MIG, SCF.

Quantikine ELISA Human CCL2/MCP1 Immunoassay (R&D Systems Inc, Minneapolis, MN, USA) was then used to measure the MCP-1 production, and Luminex Performance Assay Human MMP-12 kit (R&D Systems Inc, Minneapolis, MN, USA) to detect MMP-12.

ELISA assays were performed following the manufacturer’s instructions.

### Cell Viability (Calcein) assay

Calcein Assay was used to determine cells viability after infection and treatments and to normalize measurements from ELISA assays. 200ul of each sample was resuspended in medium without phenol red and dispensed in black 96-culture plate in triplicate. After washing, cells were incubated for 1 hour at 37° in 4 μM Calcein-AM solution (Sigma-Aldrich, St. Louis, MO, USA). Finally, fluorescence was measured at an excitation wavelength of 494 nm and an emission wavelength of 530 nm, in a plate reader. The percentage of viable cells was calculated using the following formula:% Live Cells = [*F*(530)_*sam*_–*F*(530)_*min*_] × 100%, *F*(530)_*max*_.

where F(530)_sam_ is the fluorescence at 530 nm in the experimental cell sample labeled with Calcein AM; F(530)_min_ is the fluorescence at 530 nm in a sample not labeled with Calcein AM (cell auto-fluorescence) and F(530)_max_ is the fluorescence at 530 nm in a sample where all or nearly all cells are alive, labeled with Calcein AM (sample control).

### NF-κB transcription factor assay

The activation of transcription factor NF-κB in U937 cells was determined using the NF-κB family-Transcription Factor Assay Kit (Active Motif, Carlsbad, CA, USA). Cells were plated at a concentration of 9×10^6^ cells and were harvested 18 h after M.F. infection and H_2_S treatment. Cytoplasmic and nuclear cell fractions were prepared using the Nuclear Extract Kit (Active Motif, Carlsbad, CA, USA) according to the manufacturer’s instructions. Protein concentration was determined using Bradford protein assay method and equal amounts of protein extracts (20 μg for p52 and p65, 40 μg for p50) were analyzed. The activated NF-κB subunits were detected at 450 nm with a plate reader after treatment with primary antibodies directed against either NF-κB p65, p50 or p52 subunits and followed by a secondary antibody conjugated to horseradish peroxidase (HRP), according to the manufacturer’s instructions.

### Real time quantitative RT-PCR (qRT-PCR)

U937 cells plated at a concentration of 1x10^6^ cells/well in 6 well-plates and macrophages plated in 12well-plates were used to perform the real time PCR. Cells were infected with M.F. and treated with the established concentrations of sulfide donors and then collected at 3 hours, 6 hours, 18 hours and 24 hours after the infection/treatments. RNA was extracted with the RNeasy Mini Kit (Qiagen, Frederick, MD, USA). 2 μg of RNA were reverse transcribed (iScript cDNA Synthesis Kit, BioRad, Hercules, CA, USA) and then subjected to real time PCR using the iQ SYBR Green Supermix (BioRad, Hercules, CA, USA). The cDNAs were amplified with specific primers for MCP-1 (forward, 5’-CATAGCAGCCACCTTCATTCC-3’, reverse 5’-TCTCCTTGGCCACAATGGTC-3’). Glyceraldehyde 3-phosphate dehydrogenase, GAPDH (forward, 5’-CCATGGAGAAGGCTGGGG-3’, reverse 5’-CAAAGTTGTCATGGATGACC-3’) was used as the housekeeping gene control. cDNAs were also amplified with specific primers for MMP-12 (forward, 5’-TGCACATTTCGATGAGGACG-3’, reverse 5’-GGGACTGAATGCCACGTATG-3’). Primers were selected using the NCBI/primer-blast program (http://www.ncbi.nlm.nih.gov/tools/primer-blast/) and were synthesized by Sigma-Aldrich (Sigma-Aldrich, St. Louis, MO, USA). Amplification (30 sec of denaturation at 95°C, 35 sec of annealing at 65°C and 30 sec of extension at 72°C) was performed for 35 cycles with MCP-1 and MMP-12 primers. For GAPDH primers, PCR was performed with the following protocol: 28 cycles of 30 sec at 94°C, 35 sec at 60°C and 30 sec at 72°C.

All reactions were run in triplicate. Semi-quantitative analysis was based on the cycle number (C_T_) at which the SYBR Green fluorescent signal crossed a threshold in the log-linear range of RT-PCR. The fold change in MCP-1 RNA in U937 and in infected macrophages was compared with the uninfected control at time zero of the experiment and is shown relative to the change in the expression of GAPDH RNA that was measured as an internal control.

## Results

### Mycoplasma infection induces MCP-1 in U937 cells and in human monocyte-derived macrophages

**Figure 1:**
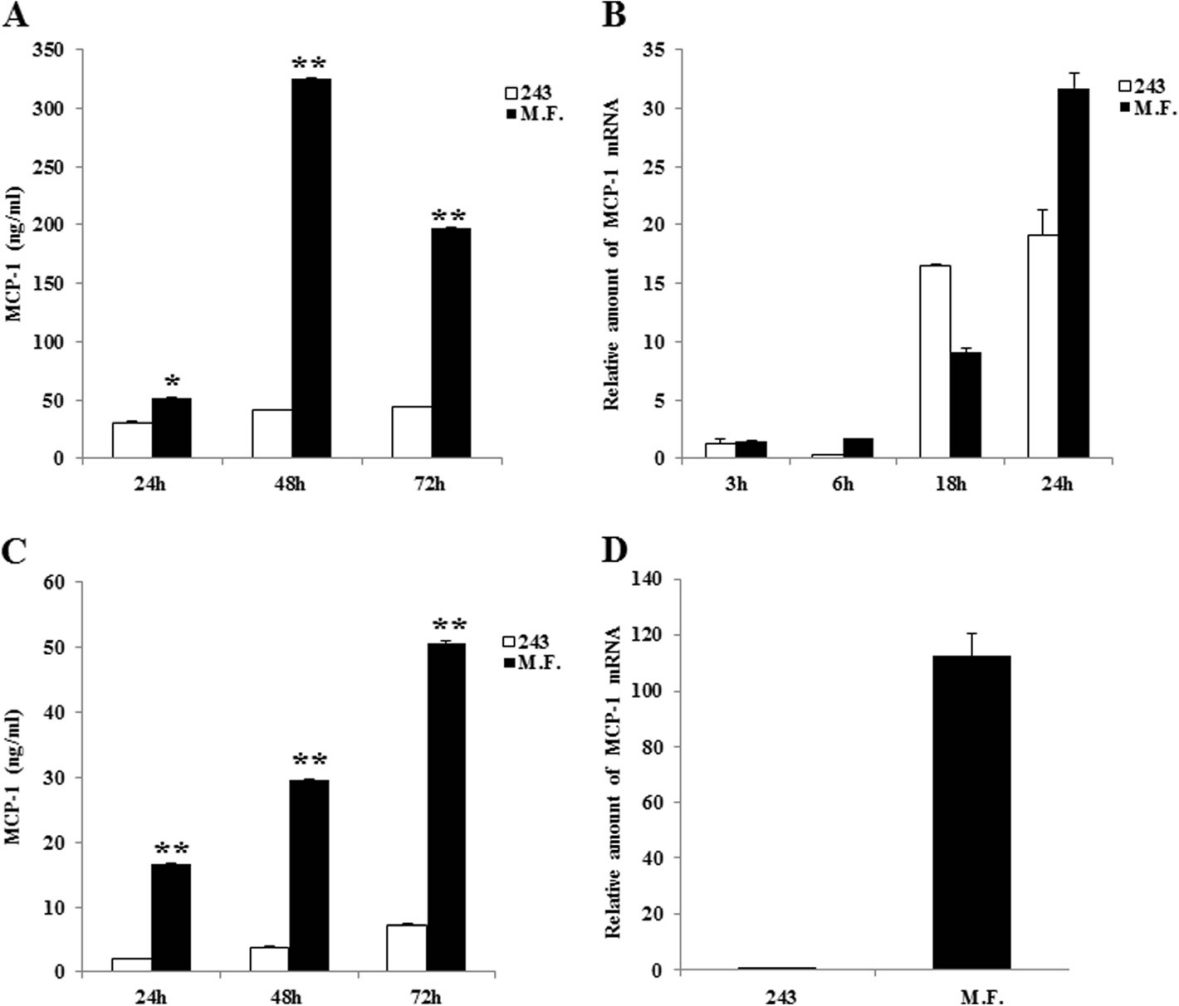
**MCP-1 induction by M.F. in U937 cells and macrophages. A**: U937 cells were infected with M.F. and the supernatants were collected at the following time points 24 h, 48 h, 72 h, and tested for MCP-1 production by ELISA. Levels of MCP-1 were indicated at each time point. **B**: RNA samples from U937 cells infected with M.F. were collected at the established time points (3 h, 6 h, 18 h and 24 h). MCP-1 expression was analyzed by real time quantitative RT-PCR as described in Materials and methods procedures. U937 cells treated with 243 M.F. growth media (v/V) were used as controls for both analysis (ELISA and qRT-PCR). **C**: Macrophages were infected with M.F. and the supernatants were tested for MCP-1 production by ELISA assay. The supernatants were collected at 24 h, 48 h and 72 h following infection. Levels of MCP-1 were indicated at each time point in M.F. infected samples and in the respective controls (uninfected cells and cells treated with 243 M.F. medium). **D**: MCP-1 expression was analyzed by real time RT-PCR in RNA samples from macrophages infected with M.F. MCP-1 relative amount of mRNA at 18 h following the infection is shown. Macrophages treated with 243 M.F. growth media (v/V) were used as controls. MCP-1 production (ELISA) was normalized with the calcein viability assay, as described in the Materials and Methods, in both experiments. Bars denote the standard deviation. The p-values were calculated as unequal variance t-test of Mycoplasma infected cells relative to 243 media control: *p ≤ 0.05; **p ≤ 0.005. The histograms shown are representative of data from three different experiments.

The induction of pro-inflammatory cytokines is a hallmark of M.F.-mediated inflammatory activity [22]. When we measured the production of cytokines and chemokines in M.F. infected cells we observed a marked increase in cytokines and chemokines production, such as MCP-1, IL-8 and IFNγ [38]. According to these results and to the role played by MCP-1 in inflammation [39], we focused our studies on this chemokine. Indeed, MCP-1 is secreted by several cells types, especially monocytes, and is responsible for the direct migration of the cells toward the endothelium at the sites of inflammation and lesion formation [30].In order to verify MCP-1 induction, macrophages and U937 cells were infected with M.F. Culture supernatants were collected at day 1, 2 and 3 following the infection and the chemokines production was measured. Uninfected cells, treated with 243 M.F. growth media (v/V), were used as controls. While basal levels of MCP-1 were produced by U937 monocytic cell line (about 40 ng/ml) [Figure 1A], infection with M.F. induced an increase in MCP-1 production with a peak 6-fold increase at 48 hours.In agreement with the ELISA data, we also observed an increase of MCP-1 mRNA both in 243 treated and M.F. infected cells, however at 24 hours the MCP-1 mRNA level in M.F. infected cells was significantly greater compared with the control (31.7-fold increase in M.F. infected cells versus 19.1-fold increase in the control) [Figure 1B].A similar effect was observed when macrophages were infected with M.F. We observed a gradual increase in MCP-1 production with peak levels at 72 hours following M.F. infection. The increase in MCP-1 production was 5.7-fold higher compared to the control [Figure 1C].MCP-1 mRNA induction following M.F. infection in macrophages was also increased at 18 hours [Figure 1D] with a 112.75-fold increase versus 0.41-fold increase in the control macrophages treated with 243 M.F. growth media.

These results suggest that M.F. induces MCP-1 expression and secretion both in U937 cells and in macrophages.

### Mycoplasma infection induces Matrix Metalloproteinase-12 (MMP-12) in U937 cells and in human monocyte-derived macrophages

**Figure 2:**
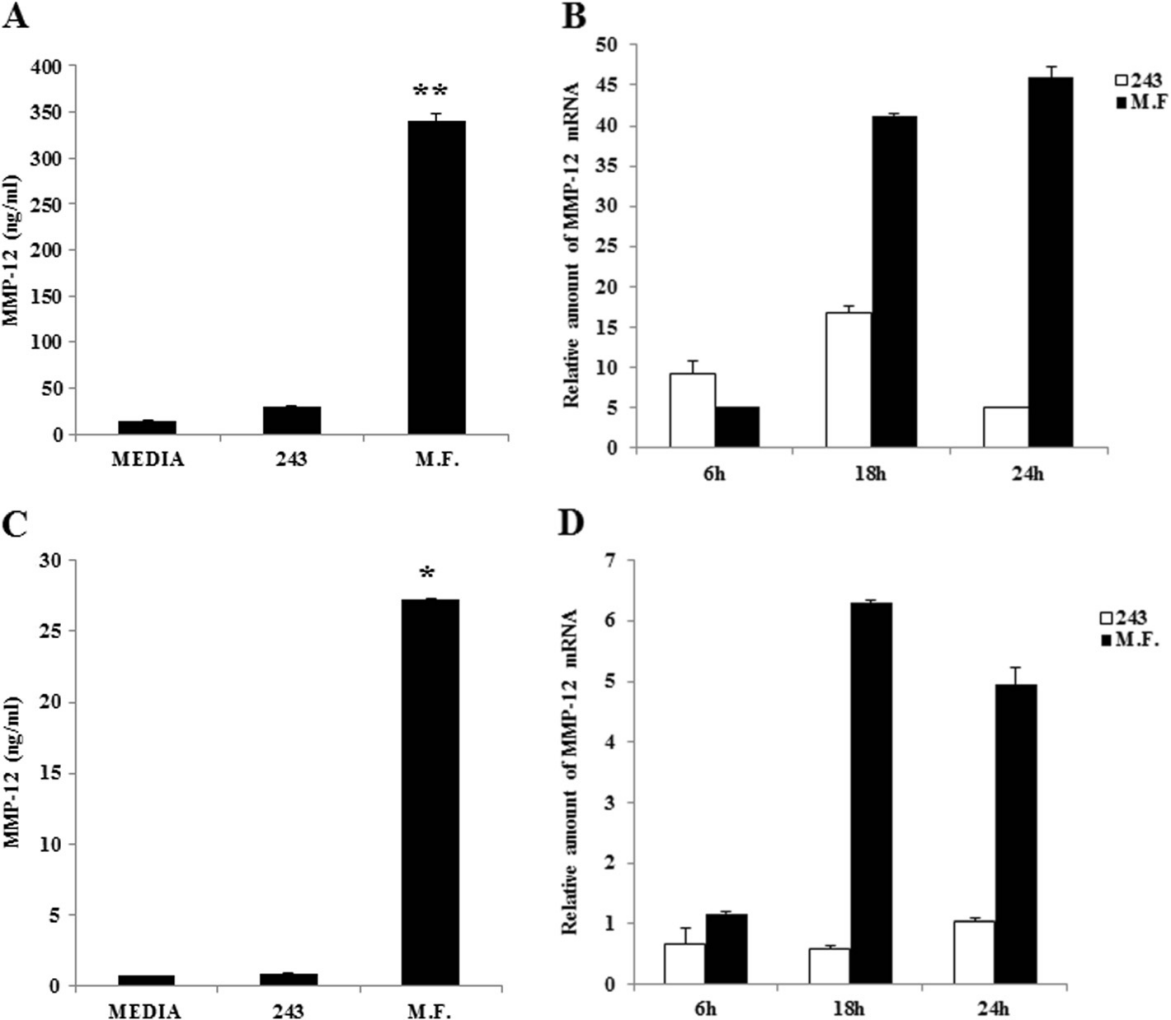
**Induction of MMP-12 mRNA and protein by M.F. in U937 cells and in macrophages.** M.F.-infected cells were analyzed by ELISA and real time PCR to detect the M.F. effects on MMP-12. **A**: 48 h following the infection, the U937 supernatants were collected and analyzed with the ELISA assay. U937 treated with 243 M.F. growth media (v/V) were used as controls for both analysis (ELISA and qRT-PCR). **B**: RNA samples from U937 cells were collected at the established time points (6 h, 18 h and 24 h) following M.F. infection. MMP-12 expression was analyzed by real time qRT-PCR as described in Materials and Methods. **C**: Macrophage culture supernatants were harvested 48 h the M.F. infection and tested by ELISA. Uninfected cells and 243 media treated cells were used as controls. **D**: RNA samples from M.F.-infected macrophages were collected at the following time points: 6 h, 18 h and 24 h. MCP-1 expression was analyzed by real time RT-PCR. Macrophages treated with 243 M.F. growth media (v/V) were used as controls. In both ELISA tests, MCP-1 production was normalized with the Calcein viability assay, as described in the Materials and Methods. Bars denote the standard deviation. The p-values were calculated as unequal variance t-test of Mycoplasma infected cells relative to 243 media control: *p ≤ 0.05; **p ≤ 0.005. The histograms shown are representative of data from three different experiments.

MCP-1 is produced as an inactive poly-protein and requires processing by MMP-12 (Matrix Metalloproteinase-12) to become functionally active [40]. Therefore we tested MMP-12 expression in U937 and macrophages infected with M.F. We measured MMP-12 levels by ELISA and we observed a marked increase of the enzyme after 48 hours (340 ng/ml) [Figure 2A]. Accordingly, U937 cells produced MMP-12 mRNA with a 16.8-fold peak of mRNA after 18 hours of culture. However, M.F. infection increased MMP-12 mRNA by 41.2-fold at 18 hours and 46-fold at 24 hours [Figure 2B].Similar to U937, macrophages showed an increase in MMP-12 secretion with a peak at 72 hours following M.F. infection (about 27 ng/ml), compared to control (about 0.76 ng/ml measured in untreated control and 0.9 ng/ml 243 media treated) [Figure 2C].Moreover, MMP-12 mRNA was increased in M.F. infected macrophages with a 6.3-fold peak after 18 hours of infection, versus 0.59-fold increase in the corresponding control. At 24 hours of culture, M.F.-infected cells had increased MMP-12 mRNA expression of 4.95-fold versus 1.04-fold in the control [Figure 2D].

Taken together these results show that MCP-1 and its activating factor MMP-12 are produced from U937 cells and macrophages infected with M.F, suggesting that MCP-1 is functionally active.

### Sulfur compounds inhibit MCP-1 production in U937 cells and in human monocyte-derived macrophages

In order to test the anti-inflammatory effect of H_2_S in M.F.-infected cells we used two different H_2_S donors: the fast-releasing H_2_S donor sodium hydrosulfide (NaHS) and the morpholin-4-ium-4-methoxyphenyl-(morpholino)-phosphinodithioate (GYY4137), a novel water-soluble molecule that, unlike NaHS, decomposes slowly to generate small amounts of H_2_S both *in vitro* and *in vivo *[41].

**Figure 3:**
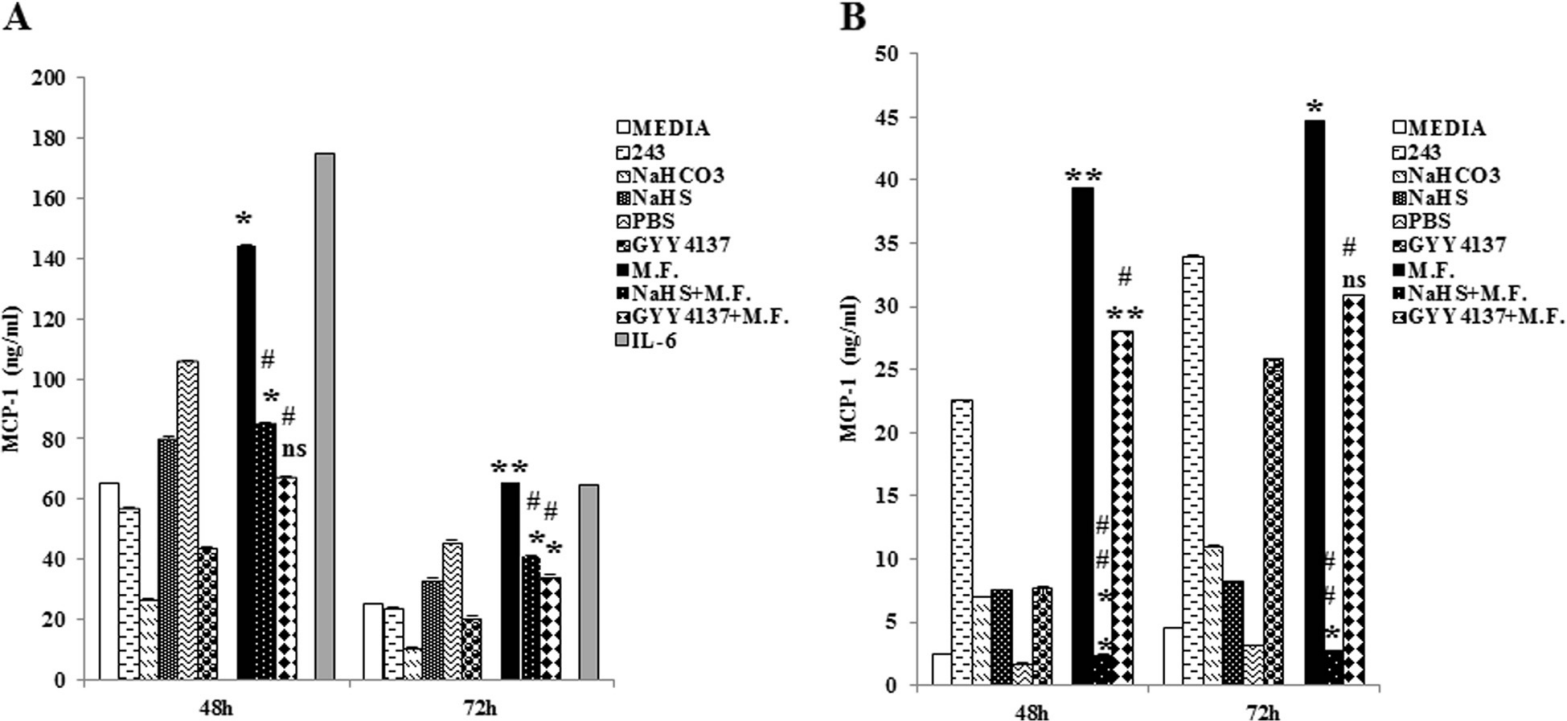
**Inhibition of MCP1 production by H**_**2**_**S in M.F. infected U937 cells and macrophages. A**: U937 cells were infected with M.F., the two H_2_S donors (NaHS and GYY4137) were directly added to the media at the concentrations of 1 mM for NaHS and 0.1 mM for GYY4137 and the supernatants were collected 48 h and 72 h after the infection/treatments, and tested for MCP-1 production by ELISA. IL-6 was used as positive control for MCP-1 production. **B**: Macrophages were infected by M.F. and treated with H_2_S donors at the same concentrations (1 mM for NaHS and 0.1 mM for GYY4137) and the supernatants were tested by ELISA assay at 48 h and 72 h following infection and H_2_S treatments. MCP-1 production was normalized with the Calcein viability assay, as described in the Materials and Methods, in both experiments. Bars denote the standard deviation. The p-values were calculated as unequal variance t-test of Mycoplasma infected cells relative to 243 media control (*p ≤ 0.05; **p ≤ 0.005; ns not significant) and as unequal variance t-test of Mycoplasma infected cells treated with H_2_S donors relative to Mycoplasma infected cells (#p ≤ 0.05; ##p ≤ 0.005). The histograms shown are representative of data from five different experiments.

NaHS and GYY4137 were directly added to the culture medium at optimal and non-toxic concentrations (see Methods) at the time of M.F infection.As expected, M.F. infection induced an increase in MCP-1 production, which was most remarkable after 48 hours of infection in U937 cells (p = 0.01) [Figure 3A] and after 72 hours of infection in macrophages (p = 0.04) [Figure 3B].Both NaHS and GYY4137 reduced MCP-1 production [Figure 3]. When U937 were infected, the effect was more marked with GYY4137 treatment compared to NaHS treatments both at 48 hours [2.1-fold reduction of MCP-1 following GYY4137 treatments (p = 0.012), versus 1.7-fold reduction of MCP-1 following NaHS treatments (p = 0.018)] and 72 hours [1.9-fold reduction of MCP-1 following GYY4137 treatments (p = 0.006), versus 1.6-fold reduction following NaHS treatments (p = 0.012)] [Figure 3A]. No changes in MCP-1 production were observed with the respective control treatments (data not shown).A similar effect was also observed when monocytes/macrophages were infected with M.F. However the amount of cytokines induced was lower compared to U937 cells, suggesting that MCP-1 production may be donor-dependent. MCP-1 was induced following M.F. infection and treatments with both NaHS and GYY4137 reduced cytokine production. Unlike U937 cells, the inhibitory effects of NaHS was more marked compared to GYY4137 treatments, both at 48 hours [1.4-fold reduction of MCP-1 following GYY4137 treatments (p = 0.012), versus 16.1-fold reduction of MCP-1 following NaHS treatments (p ≤ 0.001)] and 72 hours [1.44-fold reduction of MCP-1 following GYY4137 treatments (p = 0.02), versus 16.55-fold reduction following NaHS treatments (p ≤ 0.001)]. No changes in MCP-1 production were observed with the respective control treatments [Figure 3B].

These results highlight the anti-inflammatory effect of H_2_S in M.F.-infected cells.

**Figure 4:**
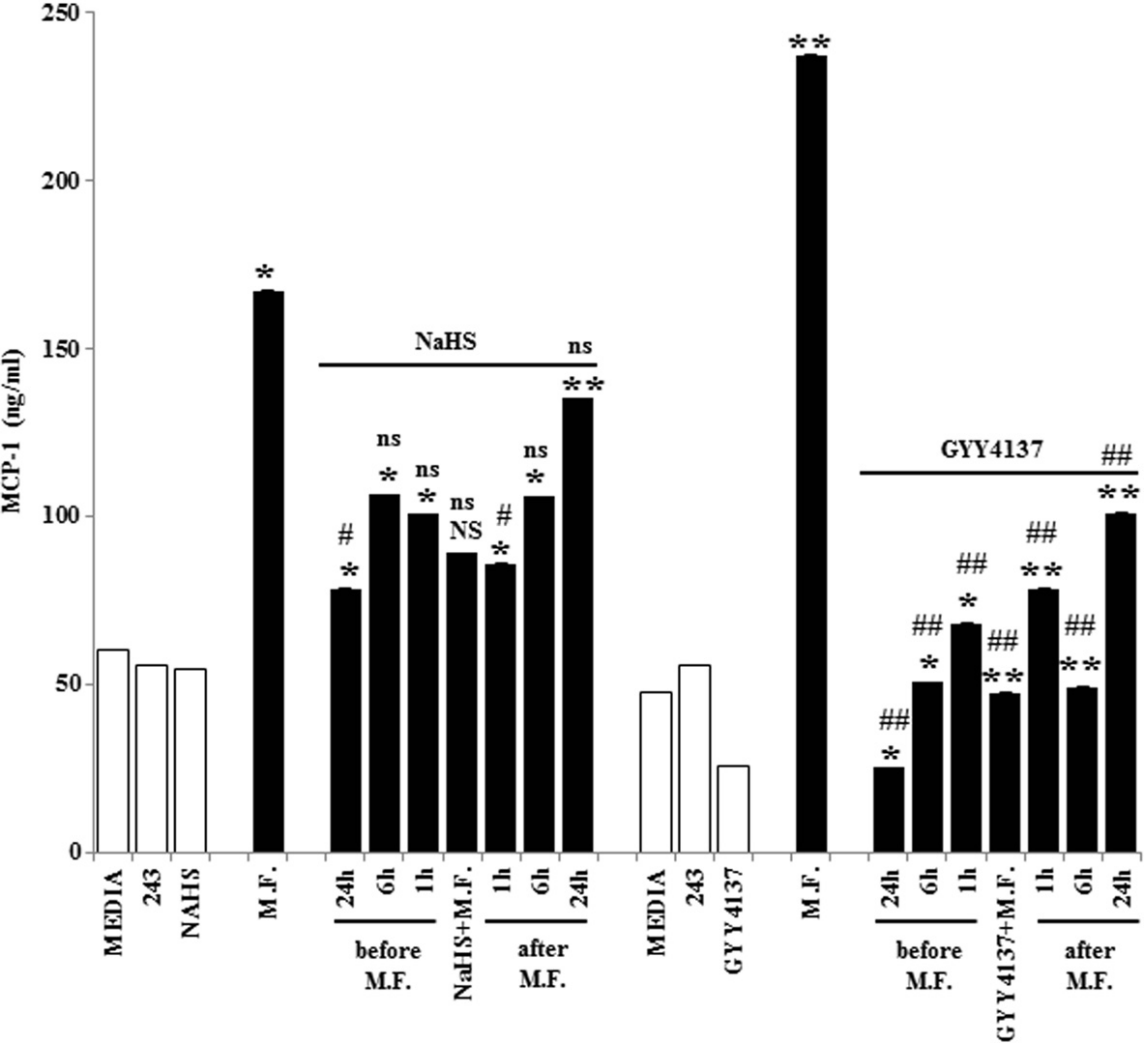
**Inhibition of MCP-1 production by H**_**2**_**S is time**-**dependent.** U937 cells were infected and treated with NaHS and GYY4137 in the following ways: at the time of M.F. infection, 1 h, 6 h, 24 h before the infection or 1 h, 6 h, 24 h after the infection. The supernatants were collected 48 h and 72 h following the infection and MCP-1 production was measured by ELISA. The chemokine production was normalized with the Calcein viability assay. 48 h time point is presented here. Bars denote the standard deviation. The p-values were calculated as unequal variance t-test of Mycoplasma infected cells relative to 243 media control (*p ≤ 0.05; **p ≤ 0.005; NS not significant) and as unequal variance t-test of Mycoplasma infected cells treated with H_2_S donors relative to Mycoplasma infected cells (#p ≤ 0.05; ##p ≤ 0.005; ns not significant). The histograms shown are representative of data from three different experiments.

We then asked the question if the time of H_2_S treatment relative to M.F. infection could affect MCP-1 production. We designed the experiments by pre-treating cells with H_2_S at 24 h, 6 h, 1 h before M.F. infection, contemporary to M.F. infection or 1 h, 6 h and 24 h following the infection. The supernatants were collected 48 h and 72 h following the infection and MCP-1 production was measured. Our data show that 1 h or 6 h of H_2_S treatment before or after M.F. infection does not affect the amount of MCP-1 production, and are comparable to MCP-1 produced when H_2_S treatment was contemporary to M.F. infection. In contrast, when U937 cells were pre-treated with H_2_S 24 h before M.F. infection, the amount of MCP-1 produced was lower compared to the contemporary treatments [2.13-fold reduction of MCP-1 following NaHS treatments (p = 0.04) and 9.42-fold reduction of MCP-1 following GYY4137 treatments (p ≤ 0.001)], suggesting that early treatments with H_2_S, and in particular with GYY4137, markedly reduce inflammation caused by M.F. [Figure 4].

Finally, when U937 cells were treated 24 h post infection there was an increase in MCP-1 production at 48 h [1.23-fold reduction of MCP-1 following NaHS treatments (p = ns) and 2.35-fold reduction following GYY4137 treatments (p = 0.001)], suggesting that at this time point the protective effect of H_2_S is reduced.

### Mycoplasma induces MCP-1 with a TLR-mediated mechanism

**Figure 5:**
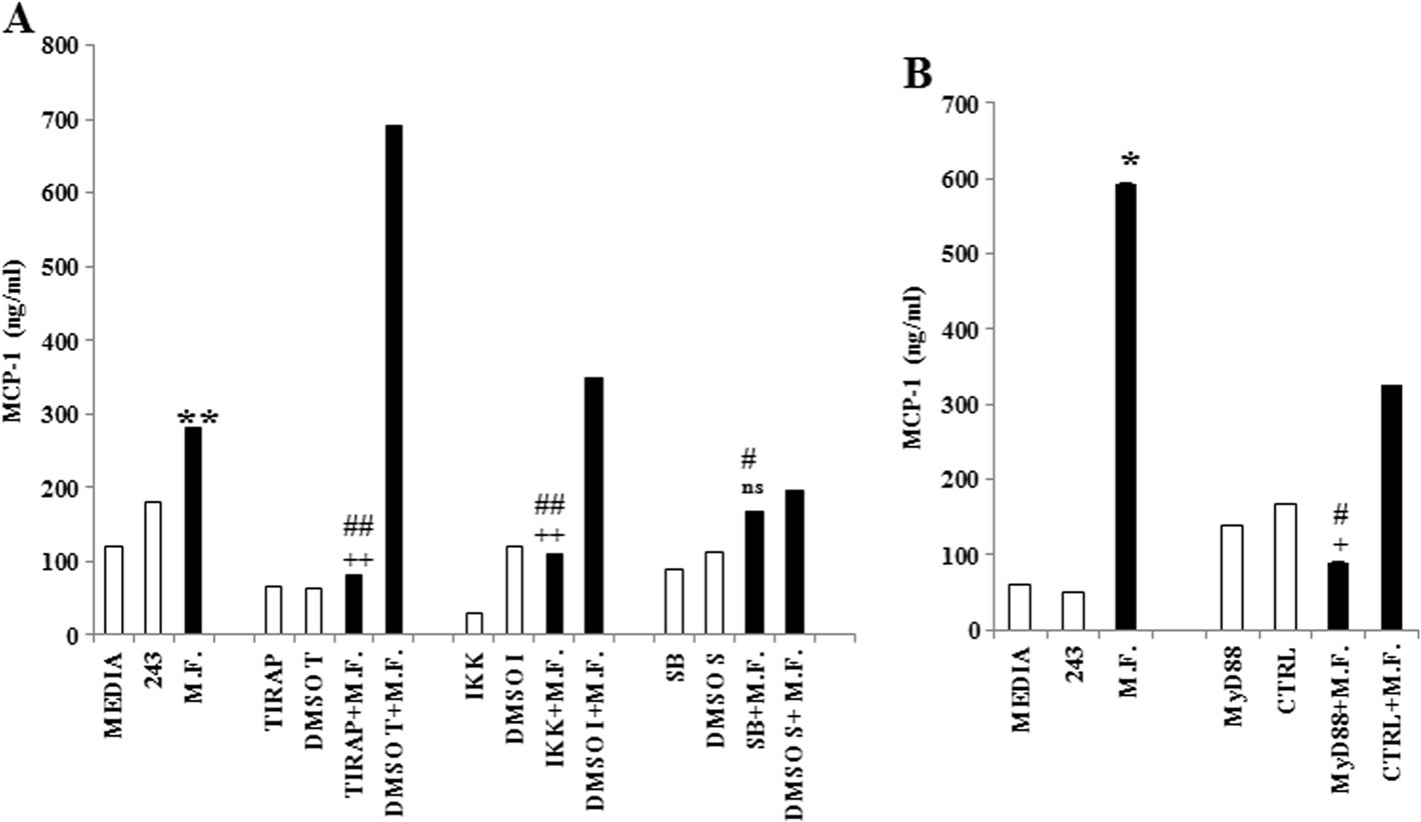
**M.F. induction of MCP**-**1 through a TLR**-**mediated mechanism.** U937 cells were infected with M.F. and treated with the following inhibitors: IKK Inhibitor VII (200nM) and TIRAP Inhibitor peptide (10 μM) to induce NF-κB inhibition **(A)**, MyD88 Inhibitory Peptide (75 μM) to inhibit MyD88 homodimerization **(B)** and SB203580 (400 μM) to inhibit p38-MAP **(A)**. The inhibitors were added daily. Supernatants were collected at 48 hours and tested for MCP-1 production by ELISA assay. MCP-1 production was normalized with the Calcein viability assay, as described in the Materials and Methods section. Bars denote the standard deviation. The p-values were calculated as unequal variance t-test of Mycoplasma infected cells relative to 243 media control (*p ≤ 0.05; **p ≤ 0.005), as unequal variance t-test of Mycoplasma infected cells treated with H_2_S donors relative to Mycoplasma infected cells (#p ≤ 0.05; ##p ≤ 0.005) and as unequal variance t-test of Mycoplasma infected cells treated with inhibitors relative to Mycoplasma infected cells treated with control inhibitors (+p ≤ 0.05; ++p ≤ 0.005; ns not significant). The histograms shown are representative of data from four different experiments.

Different signal pathways including NF-κB [42], and MAP kinases ERK1/2 and p38 [43] have been implicated in MCP-1 induction. In accordance to that, mycoplasmal lipopeptides are known to activate the NF-κB pathway [44] with a TLR-mediated mechanism, inducing pro-inflammatory cytokines.In order to define the signal pathway/s mediating M.F. induced MCP-1 production, we treated M.F.-infected cells with specific inhibitors, precisely TIRAP inhibitor peptide, IKK Inhibitor VII (IKK and IKBα inhibitors), MyD88 Inhibitory Peptide (MyD88 homodimerization inhibitor) and SB203580 (p38 MAP kinase inhibitor). Inhibitors were added directly to the cultures 1 h before infection (see Methods). Supernatants were collected 48 h and 72 h following M.F. infection and analyzed. Our results show that the specific inhibitors TIRAP and IKK Inhibitor VII [Figure 5A] were able to reduce MCP-1 production by 3.46-fold (p ≤ 0.001) and 2.57-fold (p ≤ 0.001) respectively. A more potent effect was observed after inhibiting MyD88 homodimerization, with a 6.57-fold reduction in MCP-1 production (p = 0.03) [Figure 5B]. No effect was observed with SB203580, suggesting that p38 MAPK pathway is not involved in MCP-1 induction following M.F. infection [Figure 5A]. These data suggest that M.F. induces MCP-1 expression through a TLR (toll like receptor)-NF-κB pathway.

### Sulfur compounds block MCP-1 production by inhibiting NF-κB activation

**Figure 6:**
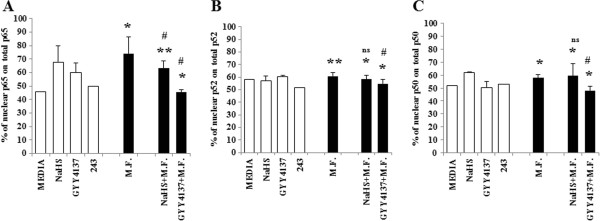
**Inhibition of NF**-**κB activation by H**_**2**_**S in M.F. infected U937 cells.** U937 cells were pre-treated with the two H_2_S donors NaHS (1 mM) and GYY4137 (0.1 mM) for 24 h. The cells were then infected with M.F. 18 h following infection the cells were collected and the cytoplasmic fraction was separated from the nuclear fraction. The percentages of NF-kB nuclear subunits p65 **(A)**, p52 **(B)** and p50 **(C)** were calculated over the total of NF-κB subunits (nuclear and cytoplasmic) detected as Absorbance (OD) reading at 450 nm. Bars denote the standard deviation. The p-values were calculated as unequal variance t-test of Mycoplasma infected cells relative to 243 media control (*p ≤ 0.05; **p ≤ 0.005) and as unequal variance t-test of Mycoplasma infected cells treated with H_2_S donors relative to Mycoplasma infected cells (#p ≤ 0.05; ##p ≤ 0.005; ns not significant). The histograms shown are representative of data from three different experiments.

In order to determine if H_2_S plays a role in NF-κB signaling, we analyzed cytoplasmic and nuclear NF-κB subunits (p65, p50, p52) during M.F. infection, with or without H_2_S [Figure 6]. We expressed the nuclear subunit as a percentages calculated over the total of NF-κB subunits (nuclear and cytoplasmic), that was detected as Absorbance (OD) reading at 450 nm.

M.F. infection caused an increase of p65 (74.1 ± 12.13% in M.F.-infected cells versus 45.5% in the control) (p = 0.01) and p50 subunits (57.9 ± 2.24% in M.F.-infected cells versus 51.7% in the control) (p = 0.02) in the NF-κB nuclear fraction, while no change was observed for the p52 subunit (p = 0.004). Treatment of the M.F.-infected cultures with H_2_S had a protective effect, particularly with the slow releasing donor GYY4137. Indeed, treatment of infected cells with GYY4137 caused a 10% reduction in the nuclear p50 subunit (p = 0.019), while NaHS had no effects. H_2_S had a more potent effect in reducing the amount of nuclear p65 subunit in infected cells: while NaHS caused 11% of reduction in nuclear p65 (p = 0.05), GYY4137 completely abrogated the increase in nuclear p65 caused by M.F. infection (45.6 ± 1.41 versus 51.7 in the control) (p = 0.009).

These results suggest that H_2_S interfere with the activation and nuclear translocation of p52 and p65 subunits, thus preventing NF-κB dimer binding to the promoters of a variety of pro-inflammatory genes.

## Discussion

There is increasing evidence showing H_2_S as a gaseous signaling molecule with a variety of effects on multiple systems. The protective effects of H_2_S, especially in the cardiovascular and in the nervous systems, are being increasingly explored. Several recent reports provide evidence suggesting a role for H_2_S in inflammation [11].

Our work highlights the anti-inflammatory effects of H_2_S during bacterial infection of macrophages with M.F.

M.F. is associated with several chronic inflammatory diseases, in particular with arthritis [24] and it has been also proposed as a putative co-factor in AIDS disease progression [45]. Multiple factors account to M.F. pathogenicity [46] and M.F. lipoproteins are known to cause innate immune response. Twenty one lipoproteins have been recently identified in the proteomics profile of MF64 [47] and among these, MALP-2 and M161 lipoproteins cause host immune activation through TLR. M161 also binds complement receptor inducing phagocytosis in macrophages and dendritic cells [23].

In our experiments, primary macrophage cultures and U937 monocytoid cell line were infected with M.F., inducing the production of pro-inflammatory cytokines; in particular MCP-1 was induced both at RNA and protein levels. The amount of MCP-1 induced in U937 cell lines (325.4 ng/ml) was constantly higher compared to the amount of MCP-1 induced in monocytes/macrophages (50.7 ng/ml) and was comparable to the MCP-1 plasma levels detected in human sepsis (2.00-128.00 ng/ml). It is known in fact that MCP-l plasma levels are detectable and increased in human sepsis and septic shock with respect to healthy patients: both surviving and non-surviving patients with sepsis or septic shock showed increased MCP-1 plasma levels compared with those of controls (<2.00 ng/ml) [48].

M.F. also induced MMP-12 that, in addition to be a pro-inflammatory molecule, is required for processing MCP-1 in its active form, suggesting that M.F.-induced MCP-1 in our systems was functionally active.

Two different H_2_S donors NaHS and GYY4137 were used to test the anti-inflammatory effects of H_2_S in M.F. infected cells. Both H_2_S donors showed anti-inflammatory activity by reducing MCP-1 production in M.F. infected cells. However while GYY4137 anti-inflammatory effect was more potent in U937 cells, NaHS was more effective in macrophages. H_2_S thus may represent a novel therapeutic molecule capable of limiting M.F.-induced inflammation, since it influences the levels of key chemokines like MCP-1 involved in the inflammatory process. Furthermore our results show that pre-treatment with H_2_S could more efficiently impact the resolution of inflammatory process.

Treatment of M.F.-infected macrophages with pharmacological inhibitors demonstrated that M.F. induces MCP-1 expression through TLR-NF-κB pathway. In particular TIRAP and MyD88 inhibitor peptides were able to completely block MCP-1 production in M.F.-infected macrophages. This pathway is essential for the activation of most pro-inflammatory genes and the constitutive activation of NF-κB pathway is associated with inflammatory diseases such as rheumatoid arthritis, bowel disease, multiple sclerosis and asthma [49].

NF-κB subunits exist in the cytoplasm in inactive forms, as a result of their association with the IkB proteins [50]. In response to a wide range of stimuli, including pathogens, stress signals and pro-inflammatory cytokines, NF-κB subunits are rapidly activated and translocated to the nucleus, assembled as NF-κB dimers which then induce the transcription of pro-inflammatory cytokines, chemokines, adhesion molecules, matrix metalloproteinases, cyclooxygenase 2 (COX2) and inducible nitric oxide synthase (iNOS) [51]. In agreement with our results obtained with the pharmacological inhibitors, M.F. infection of U937 caused nuclear translocation of p65 and p50 subunits, suggesting that M.F. infection activates the canonical NF-κB pathway. In contrast, treatment with H_2_S, particularly GYY4137, completely blocked the nuclear translocation of NF-κB heterodimer p65/p50. However NaHS only had a slight effect on NF-κB inhibition suggesting that this H_2_S fast donor might use a different mechanism to inhibit bacterial inflammation.

Our findings that H_2_S inhibits NF-κB nuclear accumulation indicate that H_2_S may target directly TLR or any of the TLR adaptors proteins, as well as signaling proteins involved in NF-κB activation. Further studies are needed to see whether a specific component of the TLR-NF-κB pathway is directly inhibited by H_2_S. Besides it is important to establish the mechanism of H_2_S inhibition of TLR-NF-κB pathway. A recent study showed that S-sulfhydration of NF-κB by H_2_S is responsible for its anti-apoptotic actions [52], therefore it will be important to verify how H_2_S-mediated protein S-sulfhydration affect the TLR-NF-κB pathway.

## Conclusions

Our data show that H_2_S inhibits the activation and the nuclear translocation of the NF-κB, reducing the transcription of pro-inflammatory genes, including MCP-1 gene.

H_2_S-releasing compounds are currently being developed as candidate drugs [3] and it is important to understand the H_2_S-mediated mechanism of action during inflammatory response to infection.

## Abbreviations

H_2_S: Hydrogen sulfide; NaHS: Sodium hydrosulfide; GYY4137: Morpholin-4-ium-4-methoxyphenyl-(morpholino)-phosphinodithioate; NF-kB: nuclear factor-kB; M.F: *Mycoplasma fermentans*; MCP-1 (CCL-2): Monocyte Chemoattractant Protein-1; MMP-12: Matrix metalloproteinase-12; TLR: Toll like receptor; MyD88: Myeloid differentiation primary response gene (88); IKK: IκB kinase; TIRAP: Toll-interleukin 1 receptor domain containing adaptor protein.

## Competing interests

The authors declare that they have no competing interests.

## Authors’ contributions

FB designed and performed experiments, analyzed data, and wrote the manuscript; SD designed and performed experiments; SK performed experiments and contributed to the writing of the manuscript; RCG provided logistic and budget support and contributed to the writing of the manuscript; GS contributed to the writing of the manuscript; DZ provided logistic and budget support and contributed to the writing of the manuscript; SC designed and performed experiments, analyzed data, and wrote the manuscript. All authors read and approved the final manuscript.
